# The association between physical activity and sleep quality among adolescents: the serial mediating roles of psychological resilience and academic stress and the moderating role of peer support

**DOI:** 10.3389/fpubh.2026.1879316

**Published:** 2026-08-10

**Authors:** Haokai Leng, Dan Ni, Meina Xiao, Fen Yu, Bicheng Ji, Zishuo Zhou, Shihong Li

**Affiliations:** 1 School of Physical Education, Shanghai University of Sport, Shanghai, China; 2 School of Physical Education, Shaoyang University, Shaoyang, Hunan, China; 3 College of Physical Education and Sport Science, Qufu Normal University, Qufu, China

**Keywords:** academic stress, adolescents, moderated mediation, peer support, physical activity, psychological resilience, serial mediation, sleep quality

## Abstract

Insufficient sleep and poor sleep quality among adolescents have become major public health concerns worldwide. Physical activity is widely associated with sleep quality, but the psychosocial factors related to this association require further examination. This cross-sectional study examined whether physical activity was indirectly associated with adolescents’ sleep quality, as indexed by Pittsburgh Sleep Quality Index (PSQI) scores, through psychological resilience and academic stress, and whether peer support moderated the association between academic stress and PSQI scores. A total of 1,347 high school students from four districts in Shanghai completed paper-and-pencil questionnaires. Physical activity, sleep quality, psychological resilience, academic stress, and peer support were measured using the Physical Activity Questionnaire for Adolescents, the Pittsburgh Sleep Quality Index, the Resilience Scale for Chinese Adolescents, the Academic Stress Questionnaire for Middle School Students, and the Peer Support subscale of the Perceived Social Support Scale, respectively. Pearson’s correlation analysis, hierarchical regression analysis, serial indirect-association analysis, and moderation analysis were conducted using SPSS 26.0 and PROCESS 4.2, with gender, grade level, household registration type, and only-child status controlled for. Higher PSQI scores indicate poorer sleep quality. Physical activity was negatively associated with PSQI scores and academic stress and positively associated with psychological resilience. Psychological resilience was negatively associated with PSQI scores and academic stress, whereas academic stress was positively associated with PSQI scores. Physical activity showed a significant direct association with PSQI scores (*β* = −0.202, 61.3%) and significant indirect associations through psychological resilience (*β* = −0.053, 16.0%), academic stress (*β* = −0.040, 12.1%), and the serial sequence of psychological resilience and academic stress (*β* = −0.035, 10.6%). Peer support moderated the association between academic stress and PSQI scores, such that this positive association was weaker among adolescents with higher peer support. These findings suggest that physical activity, psychological resilience, academic stress, peer support, and sleep quality are closely interrelated among adolescents. Given the cross-sectional design, the findings should be interpreted as statistical associations rather than evidence of temporal or causal mechanisms.

## Introduction

Insufficient sleep and poor sleep quality among adolescents have become increasingly common and are now widely recognized as public health concerns worldwide (1). Studies in China, the United States, and Canada have shown a steady decline in adolescents’ sleep duration over the past several years (2). A recent study on sleep quality among Chinese adolescents reported that 25.66% of adolescents had poor sleep quality (3). Previous research has shown that poor sleep quality is associated with adverse outcomes in adolescents’ physical and mental health (4–6), cognitive functioning (7), and academic achievement (8). Therefore, identifying factors associated with adolescents’ sleep quality is important for both theory and practice and may help inform efforts to support adolescents’ physical and mental development. As an important health behavior, physical activity is widely regarded as closely associated with sleep quality (9). Existing studies have shown that regular physical activity is associated with total sleep time, sleep efficiency, sleep onset latency, and subjective sleep quality (10, 11). From a physiological perspective, physical activity may be linked to sleep through circadian rhythm regulation, increased energy expenditure, thermoregulatory changes, and autonomic nervous system functioning (12, 13). Compared with individuals with low levels of physical activity, those who regularly engage in moderate physical activity generally have a lower risk of sleep disturbances (14). Although previous studies have supported an association between physical activity and adolescents’ sleep quality, the psychosocial factors related to this association require further examination. Existing research has rarely examined psychological resilience, academic stress, and peer support together within the relationship between physical activity and sleep quality. In the present study, sleep quality was operationalized as scores on the Pittsburgh Sleep Quality Index (PSQI), with higher scores indicating poorer sleep quality. Accordingly, the present study aimed to test a theory-driven moderated serial model of indirect associations to examine whether physical activity was indirectly associated with adolescents’ sleep quality, as indexed by PSQI scores, through psychological resilience and academic stress. It also aimed to test whether peer support moderated the association between academic stress and sleep quality. Because this study used a cross-sectional design, the proposed model should be interpreted as a theoretical and statistical association model rather than evidence of temporal or causal mechanisms.

From the perspective of positive psychological resources, psychological resilience may be closely related to both physical activity and adolescents’ sleep quality. Psychological resilience refers to an individual’s ability to adapt positively and regain balance when facing stress or adversity (15). Previous studies have suggested that physical activity is associated with better psychological adaptation among adolescents (16). Physical activity may be linked to psychological resilience through positive exercise experiences and social interaction. In turn, adolescents with higher levels of psychological resilience usually show better emotion regulation (17) and stronger stress-coping abilities (18), which may be associated with lower sleep-related psychological arousal and better sleep quality. Therefore, psychological resilience may represent an important psychological correlate in the hypothesized association between physical activity and sleep quality. However, the relationship between physical activity and psychological resilience should not be assumed to be unidirectional, as a recent systematic review indicated that resilience-related characteristics may also be associated with greater engagement in physical activity, suggesting a potentially bidirectional relationship between these variables (19). Thus, although the present study tested a theory-driven statistical association pattern linking physical activity, psychological resilience, and PSQI scores, this direction should be understood as a hypothesized statistical association rather than evidence of temporal or causal ordering. From the perspective of negative stress experiences, academic stress may also be an important correlate of adolescents’ sleep quality. Chinese high school students often face heavy academic demands, examination-oriented evaluation, and intense competition for further education (9). Higher academic stress may be associated with anxiety, tension, persistent worry, and pre-sleep cognitive arousal, all of which are closely related to poorer sleep quality (20). Physical activity may also be associated with academic stress as it may provide opportunities for emotional release, attentional diversion, and positive experiences outside academic contexts. However, this association should not be assumed to be unidirectional. Adolescents with higher academic stress may have less time, energy, or motivation to participate in physical activity. Therefore, the present cross-sectional design cannot determine whether physical activity precedes lower academic stress, whether academic stress limits physical activity, or whether both are influenced by other factors. Furthermore, as a subjective stress experience, academic stress is also related to individual psychological resources and coping abilities. Adolescents with higher levels of psychological resilience are often better able to understand and cope with academic challenges in a positive way, which may be associated with lower perceived academic stress (21). Based on this theoretical framework, the present study examined a hypothesized serial indirect-association pattern involving physical activity, psychological resilience, academic stress, and sleep quality. However, because the study was cross-sectional, the proposed serial association pattern should be interpreted as theory-driven and statistical rather than causal.

In addition, the association between academic stress and adolescents’ sleep quality may not occur independently. Instead, this association may be shaped by adolescents’ broader social support environment. Peer relationships become increasingly important during adolescence (22), and peer support may gradually become an important source of emotional comfort, stress buffering, and psychological adaptation (23). According to the stress-buffering model of social support, social support is proposed to buffer the adverse associations between stressful experiences and individuals’ physical and mental health outcomes (24). When adolescents experience high levels of academic stress, understanding, companionship, and encouragement from peers may be associated with lower tension and psychological burden. As a result, higher peer support may weaken the adverse association between academic stress and sleep quality to some extent. Therefore, peer support may moderate the association between academic stress and adolescents’ sleep quality.

In summary, although previous studies have supported associations between physical activity and adolescents’ sleep quality, the interrelations among physical activity, psychological resilience, academic stress, peer support, and sleep quality remain insufficiently understood. Accordingly, the present cross-sectional study aimed to test a theory-driven moderated serial model of indirect associations in which physical activity was associated with adolescents’ sleep quality, as indexed by PSQI scores, through psychological resilience and academic stress. It also aimed to test whether peer support moderated the association between academic stress and sleep quality. The findings may provide preliminary evidence regarding how these psychosocial factors are interrelated and may help generate hypotheses for future longitudinal and intervention studies in school settings. They should not be interpreted as evidence of causal mechanisms or as identifying definitive intervention targets. The following hypotheses are proposed:

*H1*: Physical activity is negatively associated with adolescents’ PSQI scores.

*H2*: Psychological resilience carries an indirect association between physical activity and adolescents’ PSQI scores, such that physical activity is positively associated with psychological resilience, and psychological resilience is negatively associated with PSQI scores.

*H3*: Academic stress carries an indirect association between physical activity and adolescents’ PSQI scores, such that physical activity is negatively associated with academic stress, and academic stress is positively associated with PSQI scores.

*H4*: Psychological resilience and academic stress jointly carry a serial indirect association between physical activity and adolescents’ PSQI scores, following the hypothesized statistical sequence: physical activity → psychological resilience → academic stress → PSQI scores.

*H5*: Peer support moderates the association between academic stress and adolescents’ PSQI scores, such that higher peer support weakens the positive association between academic stress and PSQI scores.

## Methods

### Participants and data collection

This study used a combination of convenience sampling and stratified random sampling. First, four districts in Shanghai, China, were selected as survey areas through convenience sampling: Hongkou District, Yangpu District, Huangpu District, and Pudong New Area. Within each district, one high school was selected as the study site after considering school type, including general high schools and vocational high schools. Next, grade level was used as the stratification criterion within each school, covering Grade 10, Grade 11, and Grade 12. Three classes were selected from each grade in each school. Since each school included three grades, nine classes were selected from each school. With approximately 40 to 45 registered students in each class, approximately 360 to 405 questionnaires were planned for distribution in each school, averaging approximately 390 questionnaires per school. Across the four schools, approximately 1,560 questionnaires were planned for distribution. Although the sample included students from multiple districts, grade levels, and school types in Shanghai, the use of convenience sampling at the district and school levels means that the sample should not be considered fully representative of the overall population of high school students in Shanghai.

The participants were high school students from the four selected districts of Shanghai mentioned above. The inclusion criteria were as follows: (20) being a high school student enrolled in one of the following districts: Hongkou District, Yangpu District, Huangpu District, or Pudong New Area, Shanghai; (25) being able to understand and complete the questionnaire independently; and (14) voluntarily participating in the study, with informed consent obtained from the student’s guardian and assent obtained from the student. The exclusion criteria were as follows: (20) having a serious physical illness, such as cardiovascular disease or an unhealed fracture, or a clinically diagnosed mental disorder, such as depression or anxiety, that could affect daily physical activity or sleep; (25) currently taking medications that may affect sleep, such as sedative-hypnotics or antidepressants; and (14) having exercise contraindications or having taken sick leave for more than 1 month due to illness. This study strictly followed the Declaration of Helsinki and the relevant requirements of the ethics committee of the authors’ institution. The ethics approval number was 102772025RT067. Before data collection, the researchers fully explained the study purpose, study content, anonymity, voluntary participation, and data confidentiality requirements to students and their guardians through the participating schools. Guardian informed consent forms were distributed through the schools and collected after being signed. In addition, an informed consent statement was presented on the first page of the questionnaire. Students who completed the questionnaire after reading and understanding the study information were regarded as having provided assent to participate. This study obtained informed consent from the guardians of the minor participants, as well as oral or written assent from the students themselves. Students received no monetary or non-monetary compensation for their participation.

Data were collected in June 2025 using paper-and-pencil questionnaires administered on site. The researchers distributed the questionnaires uniformly in the classroom and explained the instructions for completion to the students. During the survey, students completed the questionnaires independently, and the completed questionnaires were collected on site by the researchers. A total of 1,560 questionnaires were distributed, of which 1,461 were returned, yielding a response rate of 93.7%. The returned questionnaires were then screened and cleaned. After excluding 114 invalid questionnaires because of missing data, logical inconsistencies, or repetitive patterned responses, 1,347 valid questionnaires were retained, resulting in a valid response rate of 92.2%.

### Measures

#### Physical activity questionnaire for adolescents (PAQ-A)

The Physical Activity Questionnaire for Adolescents (PAQ-A) was developed by Kowalski et al. at the University of Saskatchewan, Canada. It is used to assess adolescents’ general physical activity levels during the previous week (26). The questionnaire was later introduced and adapted into Chinese by Li et al. (27). The PAQ-A includes nine items. These items mainly assess participants’ physical activity in different settings over the previous 7 days, such as physical education classes, leisure time, after school, evenings, and weekends. Each item is scored on a graded scale. The final score is calculated as the mean score of all items. A higher score indicates a higher level of physical activity. In the present study, the Cronbach’s *α* coefficient of this questionnaire was 0.826, indicating good internal consistency.

#### Pittsburgh sleep quality index (PSQI)

The Pittsburgh Sleep Quality Index (PSQI) was developed by Buysse et al. at the University of Pittsburgh in 1989. It is mainly used to assess individuals’ subjective sleep quality during the past month (28). The scale was later translated and adapted into Chinese by Liu et al. and has shown good reliability and validity in Chinese populations (29). The PSQI includes 19 self-reported items and covers seven dimensions: subjective sleep quality, sleep latency, sleep duration, habitual sleep efficiency, sleep disturbances, use of sleeping medication, and daytime dysfunction. Each dimension is scored from 0 to 3. The global score ranges from 0 to 21. A higher global score indicates poorer sleep quality. In the present study, the Cronbach’s *α* coefficient of this scale was 0.836, indicating good internal consistency.

#### Resilience scale for Chinese adolescents (RSCA)

The Adolescent Psychological Resilience Scale was developed by Hu and Gan. It was based on relevant international psychological resilience scales and the psychological developmental characteristics of Chinese adolescents (30). The scale includes 27 items and covers five dimensions: goal focus, emotion control, positive cognition, family support, and interpersonal assistance. Goal focus, emotion control, and positive cognition mainly reflect internal psychological resources. Family support and interpersonal assistance mainly reflect external support resources. The scale uses a graded scoring system. A higher total score indicates a higher level of psychological resilience. In the present study, the Cronbach’s *α* coefficient of this scale was 0.870, indicating good internal consistency.

#### Academic stress questionnaire for middle school students

Academic stress was measured using the Academic Stress Questionnaire for Middle School Students developed by Xu et al. (31). The questionnaire includes 21 items and covers four dimensions: parental stress, self-imposed stress, teacher stress, and social stress. The questionnaire uses a graded scoring system. A higher total score indicates a higher level of perceived academic stress. In the present study, the Cronbach’s *α* coefficient of this questionnaire was 0.938, indicating high internal consistency.

#### Peer support subscale of the perceived social support scale (PSSS)

Peer support was measured using the Peer Support subscale of the Perceived Social Support Scale (PSSS) developed by Zimet et al. (32). This subscale includes four items and uses a 7-point scoring system, ranging from 1 = “strongly disagree” to 7 = “strongly agree.” A higher score indicates a higher level of perceived peer support. In the present study, the Cronbach’s α coefficient of this subscale was 0.885, indicating high internal consistency.

### Control variables

To reduce the potential confounding effects of demographic factors, several variables were included as control variables in the subsequent statistical analyses. These variables included gender, grade level, household registration type, and only-child status. Gender was coded as male = 1 and female = 2. Grade level was coded as Grade 10 = 1, Grade 11 = 2, and Grade 12 = 3. Household registration type was coded as urban = 1 and rural = 2. Only-child status was coded as yes = 1 and no = 2.

### Data analysis

Questionnaire data were processed and analyzed using Excel and SPSS 26.0. First, Pearson’s correlation analysis was conducted to examine the pairwise associations among physical activity, PSQI scores, psychological resilience, academic stress, and peer support. Second, hierarchical regression analysis was performed with PSQI scores as the dependent variable. Gender, grade level, household registration type, and only-child status were entered as control variables. Physical activity, psychological resilience, and academic stress were then entered step by step to examine their associations with PSQI scores. Third, after data standardization, the hypothesized serial indirect associations were tested using Model 6 of the PROCESS 4.2 macro. The significance of the indirect associations was evaluated using bootstrap procedures with 5,000 resamples. Bootstrap 95% confidence intervals were calculated, and indirect associations were considered statistically significant when the confidence intervals did not include zero. Fourth, Model 1 of the PROCESS 4.2 macro was used to examine whether peer support moderated the association between academic stress and PSQI scores. Academic stress, peer support, and their interaction term were entered into the model to test whether the interaction term was significant. If the interaction term was significant, simple slope analysis was conducted, and an interaction plot was generated. In all statistical analyses, statistical significance was set at *p* < 0.05.

## Results

### Common method bias test

In questionnaire-based studies, common data sources and similar measurement conditions may lead to common method bias. This bias may affect the estimation of associations among variables. Therefore, Harman’s single-factor test was used to assess common method bias in the present study. All items from the measures assessing physical activity, sleep quality, psychological resilience, academic stress, and peer support were entered into an exploratory factor analysis. The results showed that the first factor explained 21.97% of the total variance, which was lower than the critical threshold of 40%. These results suggest that common method bias was not a serious concern in this study.

### Sample characteristics

Descriptive statistics for the sample were calculated using SPSS 26.0. The results are shown in Table 1. The sample included 1,347 high school students. Among them, 599 were male students (44.5%) and 748 were female students (55.5%). In terms of grade level, 517 students were in Grade 10 (38.4%), 459 were in Grade 11 (34.1%), and 371 were in Grade 12 (27.5%). Regarding household registration type, 147 students had rural household registration (10.9%), and 1,200 had non-rural household registration (89.1%). In addition, 945 students were only children (70.2%), whereas 402 were non-only children (29.8%).

**Table 1:** Sample characteristics (*N* = 1,347).

Variable	Category	*N*	Percentage (%)
Gender	Male	599	44.5%
	Female	748	55.5%
Grade level	Grade 10	517	38.4%
	Grade 11	459	34.1%
	Grade 12	371	27.5%
Household registration type	Non-rural	1,200	89.1%
	Rural	147	10.9%
Only-child status	Yes	945	70.2%
	No	402	29.8%
Total		1,347	100.0%

### Correlation analysis of the key variables

To examine the pairwise associations among physical activity, PSQI scores, psychological resilience, academic stress, and peer support in high school students, Pearson’s correlation analyses were conducted. These analyses were used to preliminarily examine the associations among the variables and to provide a statistical basis for the subsequent serial indirect-association analysis. The results are shown in Table 2. Physical activity was significantly negatively correlated with PSQI scores (*r* = −0.357, *p* < 0.01) and academic stress (*r* = −0.221, *p* < 0.01) and was significantly positively correlated with psychological resilience (*r* = 0.254, *p* < 0.01). Psychological resilience was significantly negatively correlated with PSQI scores (*r* = −0.457, *p* < 0.01) and academic stress (*r* = −0.437, *p* < 0.01). Academic stress was significantly positively correlated with PSQI scores (*r* = 0.548, *p* < 0.01). Additionally, peer support showed a significant positive correlation with physical activity (*r* = 0.108, *p* < 0.01) and psychological resilience (*r* = 0.250, *p* < 0.01) and a significant negative correlation with PSQI scores (*r* = −0.348, *p* < 0.01) and academic stress (*r* = −0.451, *p* < 0.01).

**Table 2:** Correlations among the variables.

Variable	PA	PSQI scores	PR	AS	PS
PA	1				
PSQI scores	−0.357**	1			
PR	0.254**	−0.457**	1		
AS	−0.221**	0.548**	−0.437**	1	
PS	0.108**	−0.348**	0.250**	−0.451**	1

**p < 0.01. Higher PSQI scores indicate poorer sleep quality. PA, physical activity; PR, psychological resilience; AS, academic stress; PSQI, Pittsburgh Sleep Quality Index.

### Hypothesis testing

To examine the associations of physical activity, psychological resilience, and academic stress with PSQI scores among high school students, hierarchical regression analysis was conducted. PSQI scores were used as the dependent variable. The results are shown in Table 3. In Step 1, demographic variables were entered into the model, including gender, grade level, household registration type, and only-child status. In Step 2, physical activity was added. In Step 3, psychological resilience was entered. In Step 4, academic stress was further added.

**Table 3:** Hierarchical regression analysis of the relationships among the variables.

Variable	Step 1		Step 2		Step 3		Step 4	
	*β*	*t*	*β*	*t*	*β*	*t*	*β*	*t*
Constant		1.131		7.817***		16.309***		8.316***
Gender	0.093	3.481**	0.071	2.808**	0.051	2.188*	0.037	1.753
Grade level	0.138	5.173***	0.113	4.476***	0.068	2.889**	0.017	0.788
Household registration type	0.094	3.529***	0.074	2.939**	0.047	2.019*	0.035	1.633
Only-child status	0.101	3.814***	0.076	3.033**	0.074	3.218**	0.053	2.478*
PA			−0.330	−13.087***	−0.243	−10.153***	−0.203	−9.214***
PR					−0.372	−15.437***	−0.224	−9.373***
AS							0.388	16.186***
*R* ^2^	0.058		0.165		0.291		0.407	
*ΔR* ^2^	0.054		0.161		0.287		0.403	
*F*	16.475***		44.017***		78.415***		114.734***	

*p < 0.05, **p < 0.01, and ***p < 0.001. Higher PSQI scores indicate poorer sleep quality. PA, physical activity; PR, psychological resilience; AS, academic stress; PSQI, Pittsburgh Sleep Quality Index.

In Step 1, the model including demographic variables was significant (*R*^2^ = 0.058, Δ*R*^2^ = 0.054, F = 16.475, *p* < 0.001). After physical activity was added in Step 2, the explanatory power of the model increased significantly (Δ*R*^2^ = 0.107, *F* = 44.017, *p* < 0.001). Physical activity was significantly and negatively associated with PSQI scores (*β* = −0.330, *p* < 0.001). This result indicates that higher levels of physical activity were associated with lower PSQI scores, reflecting better sleep quality. After psychological resilience was entered in Step 3, the explanatory power of the model further increased (Δ*R*^2^ = 0.126, *F* = 78.415, *p* < 0.001). Psychological resilience was significantly and negatively associated with PSQI scores (*β* = −0.372, *p* < 0.001). The negative association between physical activity and PSQI scores remained significant (*β* = −0.243, *p* < 0.001). After academic stress was added in Step 4, the model showed the highest explanatory power (Δ*R*^2^ = 0.116, *F* = 114.734, *p* < 0.001). Academic stress was significantly and positively associated with PSQI scores (*β* = 0.388, *p* < 0.001). This result indicates that higher academic stress was associated with higher PSQI scores, reflecting poorer sleep quality. At the same time, physical activity (*β* = −0.203, *p* < 0.001) and psychological resilience (*β* = −0.224, *p* < 0.001) remained significantly and negatively associated with PSQI scores.

**Figure 1:**
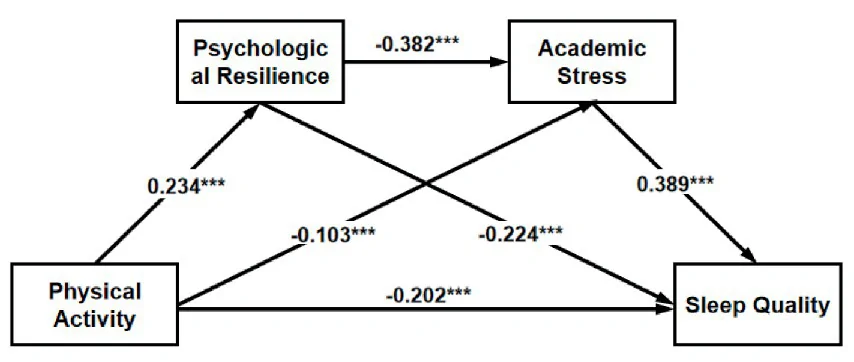
Chain mediation model of physical activity, psychological resilience, academic stress, and sleep quality (standardized coefficients). ***p < 0.001.

To further examine the relationships among physical activity, psychological resilience, academic stress, and sleep quality, a chain mediation model was constructed based on the results of the correlation and hierarchical regression analyses. This model was used to examine the indirect associations between physical activity and PSQI scores through psychological resilience and academic stress. The PROCESS macro was used to examine the hypothesized serial indirect associations. Demographic variables, including gender, grade, household registration type, and only-child status, were controlled. The main study variables were standardized before being entered into the model. The bootstrap method was used with 5,000 resamples to calculate the 95% bias-corrected confidence intervals. If the confidence interval did not include zero, the indirect association of the corresponding path was considered statistically significant. The results are shown in Table 4 and Figure 1. The direct effect of physical activity on PSQI scores was significant, with an effect size of −0.202. This effect accounted for 61.3% of the total effect. Because higher scores on the PSQI indicate poorer sleep quality, this result suggests that higher levels of physical activity were related to lower PSQI scores, that is, better sleep quality.

**Table 4:** Bootstrap test of the mediation effects.

Path	Effect	Boot SE	Boot LLCI	Boot ULCI	Proportion (%)
H1: PA → PSQI score	−0.202	0.022	−0.246	−0.159	61.3%
H2: PA → PR → PSQI score	−0.053	0.008	−0.070	−0.038	16.0%
H3: PA → AS → PSQI score	−0.040	0.010	−0.060	−0.021	12.1%
H4: PA → PR → AS → PSQI score	−0.035	0.005	−0.046	−0.026	10.6%
Total indirect effect	−0.128	0.014	−0.156	−0.100	38.7%
Total effect	−0.330	0.026	−0.381	−0.279	100%

Higher PSQI scores indicate poorer sleep quality. PA, physical activity; PR, psychological resilience; AS, academic stress; PSQI, Pittsburgh Sleep Quality Index.

For the indirect associations, all three hypothesized indirect associations were statistically significant. The bootstrap 95% confidence intervals did not include zero. First, physical activity was indirectly associated with PSQI scores through psychological resilience (H2). The indirect effect was −0.053, accounting for 16.0% of the total effect. This result indicates that higher levels of physical activity were associated with higher psychological resilience, which was in turn associated with lower PSQI scores. Second, physical activity was indirectly associated with PSQI scores through academic stress (H3). The indirect effect was −0.040, accounting for 12.1% of the total effect. This result suggests that higher levels of physical activity were associated with lower academic stress, and lower academic stress was in turn associated with lower PSQI scores.

Third, physical activity was indirectly associated with PSQI scores through the hypothesized serial sequence of psychological resilience and academic stress (H4). The indirect effect was −0.035, accounting for 10.6% of the total effect. This finding indicates that higher levels of physical activity were related to higher psychological resilience. Higher psychological resilience was further associated with lower academic stress, which was subsequently associated with lower PSQI scores. These results should be interpreted as statistical indirect associations rather than evidence of temporal or causal mediation.

### Moderation analysis

Hierarchical regression analysis was conducted to examine whether peer support moderated the association between academic stress and PSQI scores. The results are shown in Table 5. In Model 1, academic stress was significantly and positively associated with PSQI scores (*β* = 0.548, *p* < 0.001). This result indicates that higher academic stress was associated with higher PSQI scores, reflecting poorer sleep quality. In Model 2, peer support was added to the model. Academic stress remained significantly and positively associated with PSQI scores (*β* = 0.491, *p* < 0.001). Peer support was significantly and negatively associated with PSQI scores (*β* = −0.127, *p* < 0.001), indicating that higher peer support was associated with lower PSQI scores, reflecting better sleep quality. In Model 3, the interaction term between academic stress and peer support was added. The interaction term was significant and negative (*β* = −0.246, *p* < 0.001). This result indicates that peer support significantly moderated the association between academic stress and PSQI scores. Specifically, the positive association between academic stress and PSQI scores was weaker among adolescents with higher levels of peer support.

**Table 5:** Regression analysis of the moderating role of peer support.

Predictor	Outcome variable: PSQI score					
	M1		M2		M3	
	*β*	*t*	*β*	*t*	*β*	*t*
AS	0.548	24.024	0.491	19.376	0.52	21.368
PS			−0.127	−5.013	−0.097	−3.971
AS × PS					−0.246	−11.279
*R* ^2^	0.300		0.313		0.373	
Adjusted *R*^2^	0.300		0.312		0.371	

Higher PSQI scores indicate poorer sleep quality. PA, physical activity; PR, psychological resilience; AS, academic stress; PSQI, Pittsburgh Sleep Quality Index; PS, peer support.

To further clarify the moderating role of peer support, PROCESS Model 1 was used to test its role in the association between academic stress and PSQI scores. The results showed that when peer support was low (M − 1SD), academic stress had the strongest positive association with PSQI scores (*β* = 0.211, 95% CI = [0.191, 0.232]). When peer support was at the mean level (M), this positive association was weaker (*β* = 0.122, 95% CI = [0.111, 0.133]). When peer support was high (M + 1SD), this positive association was further weakened (*β* = 0.032, 95% CI = [0.014, 0.051]) (see Table 6).

**Table 6:** Test of the moderating effect of peer support.

Level of peer support		Effect	SE	95% CI lower	95% CI upper
Level	M-1SD	0.211	0.010	0.191	0.232
	M	0.122	0.006	0.111	0.133
	M + 1SD	0.032	0.009	0.014	0.051

These findings indicate that peer support moderated the association between academic stress and PSQI scores. Specifically, the positive association between academic stress and PSQI scores was weaker among adolescents with higher levels of peer support.

**Figure 2:**
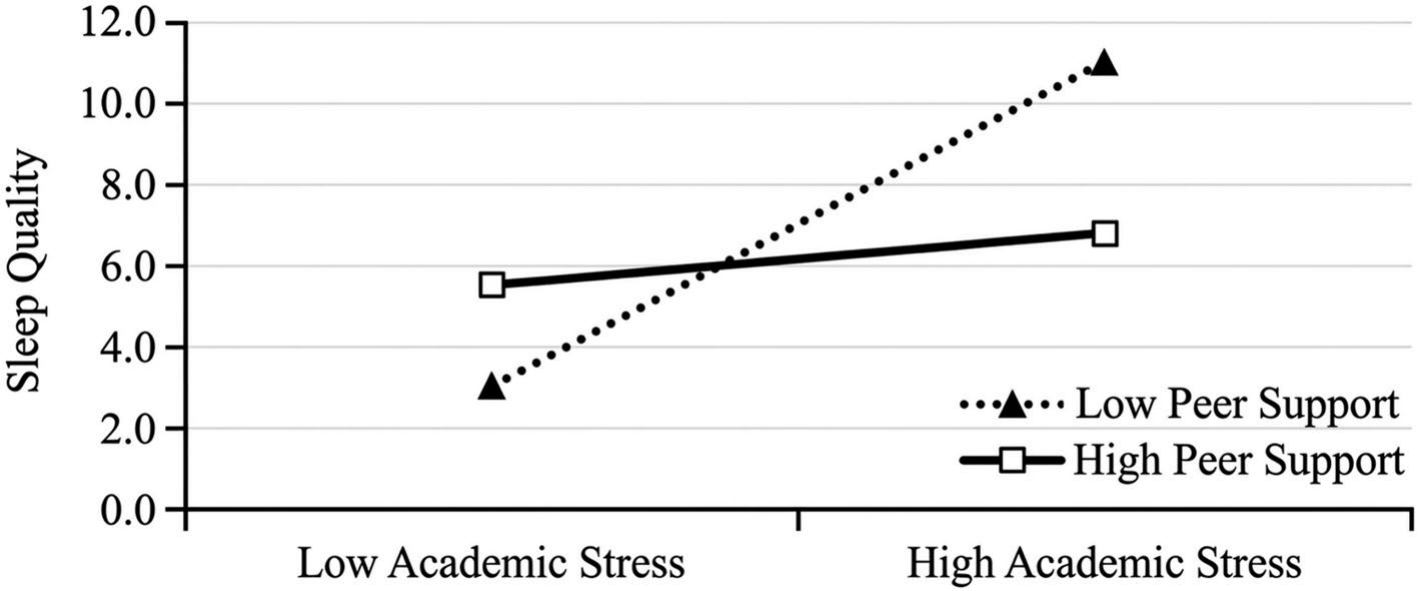
Simple slopes of the interaction between academic stress and peer support on sleep quality.

To further illustrate the moderating effect of peer support, a simple slope interaction plot was generated (see Figure 2). The results showed that peer support moderated the association between academic stress and PSQI scores. Specifically, when peer support was low, the association between academic stress and PSQI scores was stronger, as reflected by a steeper slope. In contrast, when peer support was high, this association was weaker, as indicated by a flatter slope. These findings further suggest that the positive association between academic stress and PSQI scores was weaker among adolescents with higher levels of peer support.

## Discussion

### The relationship between physical activity and adolescents’ sleep quality

The results of this study showed that physical activity was significantly and negatively associated with adolescents’ PSQI scores. Because higher total PSQI scores indicate poorer sleep quality, this negative association suggests that adolescents with higher levels of physical activity tended to report better sleep quality. This finding supports Hypothesis 1 (H1), namely, that physical activity was significantly associated with adolescents’ PSQI scores. This result is generally consistent with previous findings (33). By focusing on high school students, the present study extends previous evidence by showing that physical activity is closely associated with adolescents’ PSQI scores in this population. High school students are in a stage of rapid physical and psychological development and face heavy academic workloads as well as considerable pressure related to school advancement. As a result, their sleep quality may be particularly vulnerable to disruption. However, because physical activity was assessed over the past 7 days and sleep quality was assessed over the past month, and because the study used a cross-sectional design, this finding should be interpreted as an association between recent physical activity and past-month sleep quality rather than evidence that physical activity prospectively improves sleep quality. It is also possible that adolescents with better sleep quality are more likely to engage in physical activity or that the association is influenced by other unmeasured factors.

The observed association between physical activity and adolescents’ PSQI scores may be discussed in light of previous literature on physiological rhythms and sleep-related arousal. During adolescence, individuals’ circadian rhythms tend to show a phase delay (34). Together with factors such as academic schedules, electronic device use, and irregular daily routines, this phase delay may make adolescents more likely to experience late bedtimes, difficulty falling asleep, and disrupted sleep rhythms. Previous studies have suggested that moderate and regular physical activity may be linked to greater daytime energy expenditure, stronger nighttime sleep drive, and more stable sleep–wake rhythms (11, 12, 35). Physical activity may also be associated with lower physiological arousal before sleep and more favorable conditions for falling asleep and maintaining sleep continuity (36). In addition, the association between physical activity and adolescents’ sleep quality may also be related to psychological and social factors. Compared with adults, adolescents’ physical activity is often more situational and social in nature, frequently occurring in physical education classes, extracurricular activities, peer play, or group sports. Participation in physical activity may be accompanied by positive emotional experiences, perceived competence, self-efficacy, and peer interaction. These psychosocial factors may be associated with adolescents’ psychological resilience, academic stress, peer support, and sleep-related outcomes. Therefore, after observing the association between physical activity and PSQI scores, the present study further examined psychological resilience, academic stress, and peer support as psychosocial correlates in the hypothesized indirect-association model. These findings should be interpreted as cross-sectional association patterns rather than evidence of longitudinal mechanisms.

### The mediating role of psychological resilience in the relationship between physical activity and sleep quality

This study further examined the hypothesized indirect association between physical activity and adolescents’ PSQI scores through psychological resilience. The results showed a significant indirect association through psychological resilience, which accounted for 16.0% of the total association. This finding suggests that adolescents reporting higher levels of physical activity also tended to report higher psychological resilience, which was in turn associated with lower PSQI scores. Thus, Hypothesis 2 (H2) was supported. During adolescence, individuals’ physical and psychological development is not yet fully mature, and factors such as emotional fluctuations, environmental adaptation, and interpersonal relationships may all be associated with sleep status. However, given the cross-sectional design of the present study, this finding should not be interpreted as evidence that physical activity increases psychological resilience or that psychological resilience leads to better sleep quality.

Regarding the association between physical activity and psychological resilience, physical activity may provide adolescents with developmental contexts that differ from routine sedentary activities. Exercise and sport participation are highly process-oriented and experiential. During participation in physical activity, adolescents may experience a sense of competence, achievement, and social support through physical improvement, skill mastery, goal attainment, and teamwork. These experiences may be associated with greater self-efficacy and a stronger sense of control, which may in turn be related to more adaptive coping strategies when adolescents face difficulties or uncertain situations (37). In particular, group-based physical activity may be associated with richer social experiences through peer interaction, rule compliance, cooperation, and competition, all of which may be related to psychological resilience. Furthermore, the association between psychological resilience and PSQI scores may be interpreted in relation to emotion regulation and pre-sleep psychological arousal. Adolescents are easily affected by various events in daily life. If adolescents lack adequate psychological recovery capacity, tension, worry, or frustration generated during the day may carry over into the night and be associated with repetitive thinking before sleep, difficulty calming down, and problems falling asleep. Adolescents with higher psychological resilience may be better able to recover from negative events and show greater flexibility in interpreting difficult situations. Therefore, higher psychological resilience may be associated with lower sleep-related rumination and lower PSQI scores (38). However, the present cross-sectional data cannot determine whether psychological resilience leads to better sleep quality, whether better sleep quality supports psychological resilience, or whether both are associated with other unmeasured factors.

### The mediating role of academic stress in the relationship between physical activity and sleep quality

This study further examined the hypothesized indirect association between physical activity and adolescents’ PSQI scores through academic stress. The results showed a significant indirect association through academic stress, which accounted for 12.1% of the total association. Thus, Hypothesis 3 (H3) was supported. This finding is generally consistent with previous research linking physical activity with lower stress, lower anxiety, and better emotional states. It also indicates that the association between physical activity and adolescents’ PSQI scores may involve academic stress as an important psychosocial correlate. During high school, adolescents face heavy academic demands and intensive learning tasks. Factors such as examination performance, competition for further education, parental expectations, teacher demands, and peer comparison may all become important sources of academic stress. In the present study, higher levels of physical activity were associated with lower academic stress, and lower academic stress was in turn associated with lower PSQI scores. This suggests that academic stress may be an important psychosocial correlate in the association between physical activity and sleep quality.

From the perspective of the association between physical activity and academic stress, physical activity may provide adolescents with opportunities to temporarily disengage from academic contexts. High school students are often exposed to highly demanding learning environments for prolonged periods, during which attentional and psychological resources may be continuously depleted, and feelings of tension, worry, fatigue, and helplessness may occur. Previous research has suggested that moderate physical activity may be linked to temporary psychological detachment from ongoing academic tasks (39). Physical activity may also be associated with attentional diversion toward bodily movement, exercise goals, and activity-related contexts, which may be related to lower repetitive thinking about academic problems. At the same time, pleasurable experiences during exercise, peer interaction, and the sense of accomplishment associated with goal attainment may be linked to more positive emotional experiences and psychological relaxation. These interpretations are based on prior literature and should not be understood as causal evidence from the present cross-sectional study. Furthermore, the association between academic stress and PSQI scores may be interpreted in relation to pre-sleep anxiety and cognitive arousal. When adolescents experience high AS, they may continue thinking before sleep about unfinished homework, examination performance, educational goals, and the expectations of parents and teachers. According to Harvey’s cognitive model of insomnia, persistent worry and repetitive thinking before sleep can heighten cognitive arousal, making it difficult for individuals to enter a relaxed state and thereby increasing the risk of difficulty falling asleep and maintaining sleep (40). In the present study, lower academic stress was associated with lower PSQI scores, suggesting better sleep quality. However, the data cannot determine whether lower academic stress leads to better sleep quality, whether better sleep quality is associated with lower perceived academic stress, or whether the association reflects other unmeasured factors. Thus, the indirect association through academic stress should be interpreted as statistical rather than causal. In addition, the association between physical activity and academic stress may be bidirectional, as higher academic stress may limit adolescents’ time, energy, or motivation to participate in physical activity.

### The serial mediating roles of psychological resilience and academic stress in the relationship between physical activity and sleep quality

This study further tested a serial mediation model as a statistical model of indirect associations among physical activity, psychological resilience, academic stress, and adolescents’ PSQI scores. The results showed that the pathway “physical activity → psychological resilience → academic stress → PSQI scores” was significant, accounting for 10.6% of the total effect. This finding suggests that higher levels of physical activity were associated with higher psychological resilience; higher psychological resilience was associated with lower academic stress; and lower academic stress was in turn associated with lower PSQI scores. Thus, Hypothesis 4 (H4) was supported. However, given the cross-sectional design, this result should be interpreted as a statistical association pattern rather than evidence of a temporal or causal pathway.

From a theoretical perspective, this statistical pattern may be discussed in relation to Conservation of Resources Theory (41) and the Cognitive Appraisal and Coping Theory of Stress (42). According to Conservation of Resources Theory, individuals strive to obtain, maintain, and protect valuable resources, including personal, psychological, and social resources. Physical activity may be associated with adolescents’ physical competence, positive emotional experiences, social interaction, and perceived control. These factors may be related to psychological resilience, which may in turn be associated with how adolescents perceive and cope with academic demands. The Cognitive Appraisal and Coping Theory of Stress proposes that stress is not determined solely by external events themselves but also depends on individuals’ evaluations of environmental demands and their available coping resources. Adolescents with higher psychological resilience may have more positive cognitive appraisal styles and more adaptive coping strategies. Therefore, when facing academic demands such as examinations, homework, and competition for further education, they may be more likely to regard these demands as manageable challenges rather than uncontrollable threats, which may be associated with lower perceived academic stress. More specifically, physical activity may be linked to psychological resilience through positive experiences such as goal attainment, peer interaction, and perceived self-improvement, which may be associated with self-confidence, perceived control, and frustration tolerance. Psychological resilience may then be associated with more adaptive cognitive appraisals and coping strategies when adolescents confront academic tasks and educational pressure (43). Academic stress, in turn, may be related to pre-sleep anxiety and rumination. Higher academic stress may be associated with greater psychological arousal before sleep, whereas lower academic stress may be associated with lower PSQI scores.

In summary, the present study identified a significant serial indirect association among physical activity, psychological resilience, academic stress, and PSQI scores. Higher physical activity was associated with higher psychological resilience and lower academic stress, which were in turn associated with lower PSQI scores. These findings may help generate hypotheses for future longitudinal and intervention studies on adolescent sleep health. However, they should not be interpreted as evidence that increasing physical activity will necessarily improve sleep quality through changes in psychological resilience or academic stress. Reverse or bidirectional associations among these variables are also possible.

### The moderating role of peer support

This study further examined the moderating role of peer support in the association between academic stress and adolescents’ PSQI scores. The simple slope analysis showed that when peer support was low, the positive association between academic stress and PSQI scores was the strongest. When peer support was at moderate or high levels, this effect gradually weakened. These results suggest that peer support moderated the association between academic stress and PSQI scores. In other words, the positive association between academic stress and PSQI scores was weaker among adolescents with higher levels of peer support. Thus, Hypothesis 5 (H5) was supported.

According to the stress-buffering model of social support, social support can reduce the negative effects of stressful events on physical and mental health through emotional comfort, information provision, and the supply of coping resources (24). Previous studies have also shown that social support can alleviate the harmful effects of stress on physical and mental health by enhancing individuals’ sense of belonging, increasing coping resources, and reducing negative emotional responses (44). For adolescents, peer support is an important part of the social support system (25). Adolescence is a stage in which peer relationships become increasingly important. Peers are not only important partners in daily life but also key sources of emotional comfort, learning experience, and psychological support. More specifically, peer support may buffer the negative association between academic stress and sleep quality through both emotional support and cognitive support. On the one hand, peers often share similar learning experiences and developmental backgrounds, so they are more likely to provide empathy and understanding. Support from peers, such as companionship, listening, and encouragement, can help adolescents release the tension, anxiety, and helplessness caused by academic stress. As a result, adolescents with higher peer support may experience lower pre-sleep worry and psychological arousal. On the other hand, communication with peers may provide learning experiences, coping strategies, and positive feedback. These experiences may be associated with more adaptive views of examinations, grades, and educational competition. They may also be associated with lower rumination and threat appraisal related to academic problems. Therefore, higher peer support may be associated with a weaker adverse association between academic stress and sleep quality through lower negative emotions and pre-sleep cognitive arousal.

### Limitations

Several limitations should be noted. First, this study used a cross-sectional design, which limits the ability to determine temporal ordering or causal relationships among physical activity, psychological resilience, academic stress, peer support, and sleep quality. Therefore, the findings should be interpreted as statistical associations rather than evidence of causal mechanisms. Future studies should use longitudinal or intervention-based designs to examine the temporal relationships among these variables. Second, the measures used different reference periods. Physical activity was assessed over the past 7 days, whereas sleep quality was assessed over the past month. This mismatch limits the ability to determine whether recent physical activity preceded or influenced past-month sleep quality. Third, although the sample included students from four districts of Shanghai and covered different grade levels and school types, the districts and schools were selected partly through convenience sampling. Therefore, the sample may not fully represent the overall population of high school students in Shanghai, and caution is needed when generalizing the findings to adolescents in other regions or school contexts. Fourth, this study mainly controlled for demographic variables such as gender, grade level, household registration type, and only-child status. Other factors, such as screen time, homework duration, anxiety and depression levels, family socioeconomic status, and school schedules, were not fully controlled. In addition, all variables were measured using self-report questionnaires, which may be affected by recall bias or social desirability bias.

## Conclusion

This study showed that higher levels of physical activity were associated with lower PSQI scores among adolescents, indicating better sleep quality. Psychological resilience and academic stress showed significant indirect associations in the link between physical activity and PSQI scores, including a serial indirect-association pattern. Peer support moderated the association between academic stress and PSQI scores, such that this association was weaker among adolescents with higher levels of peer support. These findings suggest that physical activity, psychological resilience, academic stress, peer support, and sleep quality are closely interrelated among adolescents. However, given the cross-sectional design, the findings should be interpreted as statistical associations rather than evidence of causal mechanisms or intervention effects.

## Data Availability

The original contributions presented in the study are included in the article/supplementary material, further inquiries can be directed to the corresponding author.
